# Protocol for a systematic review and individual patient data meta-analysis of prognostic factors of foot ulceration in people with diabetes: the international research collaboration for the prediction of diabetic foot ulcerations (PODUS)

**DOI:** 10.1186/1471-2288-13-22

**Published:** 2013-02-15

**Authors:** Fay Crawford, Chantelle Anandan, Francesca M Chappell, Gordon D Murray, Jacqueline F Price, Aziz Sheikh, Colin R Simpson, Martin Maxwell, Gerard P Stansby, Matthew J Young, Caroline A Abbott, Andrew JM Boulton, Edward J Boyko, Thomas Kastenbauer, Graham P Leese, Matteo Monami, Matilde Monteiro-Soares, Stephen J Rith-Najarian, Aristidis Veves, Nikki Coates, William J Jeffcoate, Nicola Leech, Tom Fahey, Jayne Tierney

**Affiliations:** 1 Department of vascular Surgery, Freeman Hospital, Newcastle upon Tyne Hospitals NHS Foundation Trust, High Heaton, NE7 7DN, Newcastle, UK; 2 Centre for Population Health Sciences, Medical School, The University of Edinburgh, Teviot Place, EH8 9AG, Edinburgh, UK; 3 School of Surgical & Reproductive Sciences, 3rd Floor, William Leech Building, Faculty of Medical Sciences, Framlington Place, NE2 4HH, Newcastle upon Tyne, UK; 4 Department of Diabetes, Royal Infirmary of Edinburgh, 51 Little France Crescent, Old Dalkeith Road, EH16 4SA, Edinburgh, UK; 5 Centre for Endocrinology & Diabetes, Institute of Human Development, Core Technology Facility, room 3.30, University of Manchester, 46 Grafton Street, M13 9NT, Manchester, UK; 6 Manchester Royal Infirmary, Division of Medicine, Oxford Road, M13 9WL, Manchester, UK; 7 Epidemiologic Research and Information Center, VA Puget Sound Health Care System, University of Washington, 1100 Olive Way, Suite 1400, 98101, Seattle, WA, USA; 8 Science Consulting & Clinical Monitoring SCCM, Hessegasse 30/15, A-1220, Wien, Australia; 9 Department of Diabetes and Endocrinology, Ninewells Hospital and Medical School, DD1 9SY, Dundee, UK; 10 Medico dirigente I livello, Sezione diabetologia, Cardiologia Geriatrica, AOU Careggi, Florence, Italy; 11 Serviço de Endocrinologia–Pé Diabético, Centro Hospitalar de Vila Nova de Gaia/Espinho EPE, Unidade 1, Rua Conceição Fernandes, 4434-502, Vila Nova de Gaia, Portugal; 12 Cass Lake Indian Health Services Hospital, 425 7th St NW, MN 56633, Cass Lake, USA; 13 Harvard Medical School, 1563 Mass. Ave # 324, 02138, Cambridge, MA, USA; 14 Newcastle hospitals community health, Podiatry Department, Geoffrey Rhodes clinic, Algernon Road, Byker, NE6 2UZ, Newcastle upon Tyne, UK; 15 Department of Diabetes and Endocrinology, Nottingham City Hospital, Hucknall Road, NG5 1PB, Nottingham, UK; 16 Ward 31, Royal Victoria Hospital, NE1 4LP, Newcastle, UK; 17 Royal College of Surgeons in Ireland, 123 St Stephens Green, Dublin 2, Ireland; 18 MRC Clinical Trials Unit, Aviation House, 125 Kingsway, WC2B 6NH, London, UK

## Abstract

**Background:**

Diabetes–related lower limb amputations are associated with considerable morbidity and mortality and are usually preceded by foot ulceration. The available systematic reviews of aggregate data are compromised because the primary studies report both adjusted and unadjusted estimates. As adjusted meta-analyses of aggregate data can be challenging, the best way to standardise the analytical approach is to conduct a meta-analysis based on individual patient data (IPD).

There are however many challenges and fundamental methodological omissions are common; protocols are rare and the assessment of the risk of bias arising from the conduct of individual studies is frequently not performed, largely because of the absence of widely agreed criteria for assessing the risk of bias in this type of review. In this protocol we propose key methodological approaches to underpin our IPD systematic review of prognostic factors of foot ulceration in diabetes.

Review questions;

1. What are the most highly prognostic factors for foot ulceration (i.e. symptoms, signs, diagnostic tests) in people with diabetes?

2. Can the data from each study be adjusted for a consistent set of adjustment factors?

3. Does the model accuracy change when patient populations are stratified according to demographic and/or clinical characteristics?

**Methods:**

MEDLINE and EMBASE databases from their inception until early 2012 were searched and the corresponding authors of all eligible primary studies invited to contribute their raw data. We developed relevant quality assurance items likely to identify occasions when study validity may have been compromised from several sources. A confidentiality agreement, arrangements for communication and reporting as well as ethical and governance considerations are explained.

We have agreement from the corresponding authors of all studies which meet the eligibility criteria and they collectively possess data from more than 17000 patients. We propose, as a provisional analysis plan, to use a multi-level mixed model, using “study” as one of the levels. Such a model can also allow for the within-patient clustering that occurs if a patient contributes data from both feet, although to aid interpretation, we prefer to use patients rather than feet as the unit of analysis. We intend to only attempt this analysis if the results of the investigation of heterogeneity do not rule it out and the model diagnostics are acceptable.

**Discussion:**

This review is central to the development of a global evidence-based strategy for the risk assessment of the foot in patients with diabetes, ensuring future recommendations are valid and can reliably inform international clinical guidelines.

## Background

Diabetes–related lower limb amputations are associated with considerable morbidity and mortality and are usually preceded by foot ulceration. A recent analysis of diabetes-related amputation rates in England found there is a wide variation in incidence and researchers suggest that this may be explained by a variation in the delivery of care [1]. Annual assessment procedures are recommended to identify those people with diabetes who are at risk of foot ulceration [2-5] and there is some evidence to support the use of certain diagnostic tests, symptoms and signs but the role of other contributory factors is less clear [6]. Currently there is insufficient evidence that interventions to reduce foot amputations or ulcerations are clinically or cost effective [7].

Meta-analyses based on aggregate data of independent prognostic factors found the duration of diabetes, HbA1c, Peak Plantar Pressure (PPP), and vibration perception threshold (VPT) all distinguish between those people who will develop a foot ulcer and those who will not. However, there was significant heterogeneity between studies which may be due to differences in lengths of follow-up, methods of ascertaining the presence of ulcers and the use of different cut-off points (thresholds) for some of the tests [6].

A systematic review of clinical prediction rules (CPRs) for assessing the risk of developing diabetic foot ulceration in people with diabetes identified five different risk stratification tools derived from consensus amongst clinical experts, literature reviews and prospective studies using logistic regression methods [8]. The prognostic factors were foot deformity, peripheral neuropathy, peripheral vascular disease (pulses and/or ABI), and previous amputation, the presence of callus, the HbA_1_c, Tinea pedis, and onymychosis. The review concludes that the CPR which possesses the greatest accuracy continues to be the subject of debate.

Both reviews [6,8] found wide variations in the estimates of the incidence of foot ulceration across different study populations (2% to 17%) and as prediction tools derived from high risk populations may be of less value in the general diabetic population, this deserves further consideration.

These systematic reviews represent the best attempts to integrate evidence of prognostic factors to date, [6,8] but the findings are compromised because authors of some primary studies report adjusted estimates whilst others report unadjusted estimates and it is unclear whether the same confounders or effect modifiers have been used. Conventional meta-analytic techniques using data that have been estimated or averaged across all individuals in a study - aggregate data - do not permit adjustments for confounding to be performed and the best way to reliably analyse data from several cohort studies using a standard approach is to use individual patient data (IPD) [9,10].

There are several advantages of IPD meta-analyses, but the ability to conduct a more complete time-to-event analysis, where outcomes can be adjusted for prespecified covariates is key. We propose to conduct a systematic review and meta-analysis of IPD to create a statistical model of independent predicative factors of diabetic foot ulceration [9-11].

A detailed assessment of 20 IPD articles conducted as part of a synthesis of IPD reviews identified many challenges associated with this research method and the time-consuming and costly nature requires proper consideration. Fundamental methodological omissions are common: the assessment of the risk of bias arising from the conduct of individual studies is not usually performed and *a priori* study protocols and ethics are rare [12].

Our protocol has been informed by this work and we have identified 15 cohort studies through searches of electronic databases. These cohorts include data from approximately 17,000 patients worldwide and the authors of the original reports have agreed to collaborate in this research by sharing data.

This review is central to the development of a global evidence-based strategy for the risk assessment of the foot in patients with diabetes, ensuring future valid recommendations that can reliably inform international clinical guidelines. A flow diagram of the stages involved in a systematic review of IPD can be found in Additional file 1: Appendix 1 [10].

## Presentation of the hypothesis

### Planned investigation

#### Research questions

•What are the most highly prognostic factors for foot ulceration in people with diabetes (symptoms signs, diagnostic tests and elements from the patient history) based on IPD analysis?

•Can the data from each study be adjusted for a consistent set of adjustment factors?

•Does the model accuracy change when patient populations are stratified according to demographic or clinical characteristics?

## Testing the hypothesis and Implications of the hypothesis

### Planned investigation

#### Research objectives

We will systematically review cohort studies and include individual patient data in a meta-analysis to estimate the prognostic value of clinical characteristics and diagnostic test results. This will allow us to develop a prognostic model of the risk factors for diabetic foot ulceration (DFU) based on data collected worldwide. We will test the robustness of the model in different demographic profiles – for example, age, duration of diabetes, control of diabetes (insulin, diet or oral medication) and type of diabetes (Type I, Type II).

### Search method

The electronic search strategies used for in a previous systematic review by members of our group will be conducted according to the published methods [13]. Copies of the EMBASE and MEDLINE search strategies can be found in Additional file 2: Appendix 2.

### Selection criteria

One reviewer will apply the IPD review eligibility criteria to the full-text articles of the studies identified in our literature search and also all studies excluded from our aggregate systematic review to ensure we do not miss eligible IPD. A second reviewer will apply the eligibility criteria to a 10% random sample of the abstract search yield to check that no relevant material will be missed by having only one reviewer assess all the abstracts.

### Eligibility criteria

#### Types of Participants

The IPD review will only include data from individuals who are free of foot ulceration at the time of study entry and who have a diagnosis of diabetes mellitus (either type 1 or type 2). Corresponding authors of all identified cohort studies will be contacted and invited to share their data. When we identify studies with patients who had prevalent foot ulcers at the time of recruitment, we will ascertain whether IPD are available for patients who were free of ulceration at the time of recruitment.

#### Types of exposure variables

All elements from the patient history, symptoms, signs and diagnostic test results will be considered for inclusion in the prognostic model. These are collected variously as continuous, binary and multi-categorical data.

#### Type of outcome variable

The outcome variables will be incident foot ulceration (present/absent) and time to ulceration from initial diagnosis of diabetes as well as from the time of screening.

#### Types of studies

We will seek data from all cohort studies which included participants who were free of foot ulceration at the time of study recruitment. Our previous work indicates that data collected in older studies could be difficult to obtain and we are aware that some investigators are no longer in possession of their study data (Personal communication, D. Armstrong 2012). Where data are unavailable, details of the study will be presented in aggregate form in the final report.

Cohort studies which recruited patients with prevalent and incident foot ulceration will be considered for inclusion where it is possible to separate the data for these patients.

### Data extraction and quality assessment

Data extraction will be undertaken by 2 reviewers working independently and disagreement will be resolved by discussion. For quality assessment, a 2-stage process will be used; 2 reviewers working independently will complete those items available from the published report together with information provided by authors of the primary studies.

The assessment of methodological quality is an important component of an IPD systematic review but there is complexity in assessing potential threats to the validity of primary studies for this research genre. No widely agreed criteria exist for assessing the risk of bias in aggregate systematic reviews of prognostic studies [14] and there is a complete absence of established guidelines for prognostic IPD reviews (personal communication, D. Altman, R.Riley 2012). Although flaws in the recruitment of patients or the manner of data collection can influence review findings, some domains usually assessed by systematic reviewers of published reports are irrelevant, e.g. those pertinent to the analysis performed by the primary authors. We have compiled a list of items relevant to our IPD review question which are likely to identify studies with data which are compromised by threats of validity. This checklist of items can be found in Additional file 3: Appendix 3 [15-26], it has been refined during a pilot phase by 2 researchers working independently.

### Heterogeneity

As with any meta-analysis, heterogeneity must be considered, both from a clinical and statistical viewpoint. First, clinical expertise will be used to decide if it would be meaningful to combine the studies based on the patient demographics, risk factors (symptoms, signs and diagnostic test results), outcome measures and timing of outcome measures (length of follow-up). We will examine histograms of relevant variables from each dataset to check the spread, mean, median, and skewedness, and the consistency of these properties across datasets, before reaching a decision about whether it makes clinical or statistical sense to combine the data. We will also consider relationships between variables using tables and scatter plots.

Sources of heterogeneity that particularly concern us are differences between the patient groups with regard to basic demographics and disease spectrum as these may have a strong influence on prognosis and the performance of the tests. Also important are the various methods used to conduct the tests, which again may lead to marked differences in test performance. Another potentially important source of heterogeneity is length of follow-up as this may impact on the proportion of patients who develop ulceration. These aspects will be carefully detailed during the review process.

We are aware that a consensus has not yet been reached about the investigation of heterogeneity in IPD systematic reviews. Therefore we will use conventional methods of investigating heterogeneity on aggregate data generated from the datasets. We shall therefore generate summary measures and use these to create forest plots and compute I [2] statistics [27]. I [2] values of 50% and 75% have been used to denote moderate and high levels of variation between studies that are not explainable by chance. We shall use these figures as a guide only, together with the results from the IPD [28].

### Statistical analysis

We propose to use a multi-level mixed model, using “study” as one of the levels. Such a model can also allow for the within-patient clustering that occurs if a patient contributes data from both feet, although to aid interpretation, we prefer to use patients rather than feet as the unit of analysis. We will only attempt this analysis if the results of the investigation of heterogeneity do not rule it out and the model diagnostics are acceptable.

As the datasets should contain the date of initial diagnosis of diabetes and the date, if any, of foot ulceration, we propose to use survival analysis. Covariates will be added to the model based on clinical relevance, if there are many possible covariates that could be added given the number of events and patients and there is a danger of model over-fitting, the clinicians will be asked to choose a subset of covariates based on their expertise and experience. We shall not use data-derived methods as these lead to overly optimistic estimates of model performance. Model performance will be assessed graphically and with chi-square and other goodness-of-fit statistics.

As we plan to use the patient, rather than the foot, as the unit-of-analysis, we can use a simpler model that will be easier to interpret. It is also important from the view of patient outcomes – an amputation affects the patient as a whole and not just the foot. One approach to construct the model is to use the most badly affected foot from each patient. However, if the model performance merits an analysis using the foot as the unit-of-analysis, and of course allowing for the correlation between feet belonging to the same patient, we shall conduct such an analysis.

To avoid a loss of information, wherever possible we shall keep continuous variables as continuous and not dichotomise or otherwise categorised variables, e.g. we shall use BMI, rather than subdivide patients into “underweight”, “normal weight”, “overweight”, and “obese”. Sometimes the relationship between a continuous covariate and the outcome is not linear, and in such cases we will investigate the use of fractional polynomials and similar.

### Validation of the dataset

We intend to undertake both internal and external validation of the prognostic model. For internal validation, we will not divide the datasets into development and validation subsets, as this is a relatively inefficient method of validating prediction models. Instead we shall use bootstrapping as it is less susceptible to bias and leads to more stable model development [28]. For external validation, we shall reserve one or two of the datasets to test the final model obtained in the main analyses. The reserved datasets will be chosen on the basis of completeness of variables collected so that, we hope, all the variables present in the final model will also be in the reserved datasets, thus requiring no or minimal modification of the final model for external validation purposes. We also shall look at various characteristics of each dataset such as patient demographics when choosing the reserve datasets to ensure that these datasets are not atypical of the set of datasets.

Unfortunately we are currently lacking the data required for a full power calculation. However, as an illustration, assuming that it is possible to split the sample of 17000 evenly in half into patients with and without some prognostic factor, it would be possible to detect a 2% difference in the proportions of patients with foot ulcers in each group with over 90% power. This calculation assumes that the ulceration rate in one group is 0.10 and 0.08 in the other. With a Type I error rate of 0.05, these figures give a power of 99.53%.

### Handling missing data

Our method for handling missing data will depend on the extent of the missingness and if the mechanism causing the missingness is known, specifically if they are missing completely at random, or not. If the datasets contain missing data for which there is no explanation, they will be assumed to be ‘missing at random [29].

We will use ICE multiple imputation (ICE programs, Stata 11.0) [30], and include all available patient variables (including the patient outcome: foot ulceration) in the imputation model to help predict missing data for the variables of interest. Twenty imputed datasets will be used and included in the imputation procedure. To test the validity of the imputation, a sensitivity analyses will be performed restricting our cohort to patients without missing data (complete case analysis) [31].

### Specifying variables for analysis

A full list of the most common variables reported in cohort studies is presented in Additional file 4: Appendix 4. Examples of variables of interest are below. Importantly the dates relating to patient recruitment, the timing of the measurement of variables and the date of follow-up are also required.

Continuous variables (and date measured)

1. Age

2. BMI

3. HbA1c

4. ABI

5. Peak plantar pressure (PPP)

6. Duration of diabetes

Binary and other categorical variables (and date measured)

1. Gender

2. Cutaneous sensation (monofilaments)

3. Vibration Perception Thresholds (VPT (tuning forks and neuro or biothesiometers))

4. Absent pedal pulses

5. Diabetes-related medication use

Outcome variable; Incident foot ulceration (present/absent) and time to ulceration (date measured).

### Supplying the data

The authors of the cohort studies will be able to supply data in any way that is most convenient to them. A single individual will be identified for each study to whom all queries about the data collection processes and transformation of individual variables will be addressed. The research committee structures can be found in Additional file 4: Appendix 4.

### Ethics and governance

The ethics of obtaining data collected from a number of sources which cross international boundaries and different legal systems have been carefully considered and informed by ethics advice issued by the Medical Research Council (UK). This study does not require separate ethical committee approval for the following reasons;

•Investigators of each of the original studies obtained local ethical committee approval and written, informed patient consent prior for each of the cohorts included in the IPD review.

•The project seeks anonymised data from which the individuals recruited to the original study cannot be identified [32].

The value of the IPD analysis will be the production of a global dataset of prognostic factors for diabetic foot disease and the opportunities for new uses will be maximised. Anonymised data from each of the collaborators of the primary cohort studies will be transported in a manner deemed most convenient to original study investigators including encrypted USB sticks if required.

Data will then be formatted in a consistent way to permit a re-analysis. Data will be stored in password protected files on a secure University of Edinburgh computer [University of Edinburgh Data protection registration number: [Z6426984]] and will only be accessible by a member of the Data Management Committee, membership of which can be found in the appendices.

This protocol incorporates a data confidentiality agreement which makes clear the need for the data provided to de-identify individual patients. It also includes an assurance that the original investigators are in possession of local ethical approval for their study.

### Communication

Regular e-mail updates will be used to inform the international group of our activities. Electronic media such as Drop Box and e-mail may be used to store and exchange data and paperwork between the original investigators and the researchers. When researchers are cleaning a specific data set they may communicate with the original investigators via telephone discussions or by email.

### Collaborators face to face meeting

Once the initial analysis has been performed, a face-to-face meeting of all collaborators will be convened. The purpose of the meeting is to allow the collaborators know the results of the review and meta-analysis first and to have the opportunity to interpret the data and question the findings Additional file 5: Appendix 5 and Additional file 6: Appendix 6.

### Reporting

In the final report we will clearly present the methods of the review such as tabulated characteristics of included studies and details of study designs. The report will conform to recommendations in the PRISMA checklist. Formal synthesis of the results and formal assessments of study quality will also be presented [33].

This protocol is registered with PROSPERO (International Prospective Register of Systematic Reviews) at the NHS Centre for Reviews and Dissemination (CRD) at the University of York [34]. [Registration number: CRD42011001841].

### Public Partners Involvement (PPI)

The research is supported by a public partner from Diabetes UK who ensures the research incorporates aspects of risk assessment that matter to patients. His views, opinions and perspective have ensured the study documentation and data collection processes are acceptable to the general diabetic population.

## Competing interests

The authors have no financial competing interests.

## Authors’ contributions

FC is the Chief Investigator, and GDM the Principal Investigator with overall responsibility for the statistical analysis, together with CA and FMC they form the Data Management Committee and have day to day responsibility for the research. Together with JFP, AS,CRS, GPS,MJY and MM they form the research steering committee each of whom are co-investigators and have contributed to the design and writing of the research protocol. CAA,AJMB,EJB,TK,GPL,MM,MM-S,SJR-N,AV are the principal investigators/corresponding authors on all cohort studies identified by the review search strategy. They have contributed individual patient data to the meta analysis. Together with the Research Steering Committee and the independent advisors they form the international steering committee work closely with members of the data management committee in the preparation of the data. NC,WJJ,NL,TF,JT are the project independent advisors who make clinical and methodological contributions. They have collaborators status and are members of the International Steering committee. All authors read approved the final manuscript.

## Research funding

This project was funded by the National Institute for Health Research Health Technology Assessment (NIHR HTA) Programme (project number 10/57/08) and will be published in full in Health Technology Assessment.

The views and opinions expressed therein are those of the authors and do not necessarily reflect those of the HTA programme, NIHR, NHS or the Department of Health.

## Pre-publication history

The pre-publication history for this paper can be accessed here:

http://www.biomedcentral.com/1471-2288/13/22/prepub

## Supplementary Material

Additional file 1: Appendix 1 Flow diagram of the stages in an IPD review adapted from Stewart and Clark 1995^10^. Click here for file

Additional file 2: Appendix 2 Embase and MEDLINE searches. Click here for file

Additional file 3: Appendix 3 Questionnaire to determine the methodological standards adopted in cohort studies evaluating the prognostic factors for foot ulceration in diabetes. Click here for file

Additional file 4: Appendix 4 List of the most common variables reported in cohort studies. Click here for file

Additional file 5: Appendix 5 Committees and members. Click here for file

Additional file 6: Appendix 6 Data agreement for the collaborators. Click here for file

## References

[B1] Holman N Young RJ Jeffcoate WJ Variation in the recorded incidence of amputation of the lower limb in England Diabetologia 2012 55 7 1919 25 10.1007/s00125-012-2468-6 22398645

[B2] General Medical Services Contracthttp://www.nice.org.uk/aboutnice/qof/indicators_detail.jsp?summary=13080 Checked 24/04/2011

[B3] McIntosh A Peters J Young R Prevention and management of foot problems in type 2 diabetes: clinical guidelines and evidence 2003 Sheffield: Sheffield University NICE guideline 21698845

[B4] The International consensus on the diabetic foot. In International concensus on the diabetic foot 2012 http://iwgdf.org/ [accessed 28/09/2012

[B5] Scottish Intercollegiate Guideline Network The Management of Diabetes. A National Clinical Guideline (No 116) ISBN 978 1 905813 58 2 March 2010. http://www.sign.ac.uk/guidelines/fulltext/116/index.html (accessed 22/01/2013)

[B6] Crawford F Inkster M Kleijnen J Fahey T Predicting foot ulcers in patients with diabetes: a systematic review and meta-analysis Q J Med 2007 100 2 65 86 10.1093/qjmed/hcl140 17277315

[B7] Dorresteijn JAN Kriegsman DMW Valk GD Complex interventions for preventing diabetic foot ulceration Cochrane Database Syst Rev 2010 1 CD007610 10.1002/14651858.CD007610.pub2 20091642

[B8] Monteiro-Soares M Boyko EJ Ribeiro J Ribeiro I Dinis-Ribeiro M Risk stratification systems for diabetic foot ulcers: a systematic review Diabetologia 2011 54 5 1190 1199 10.1007/s00125-010-2030-3 21249490

[B9] Riley RD Lambert PC Abo-Zaid G Meta-analysis of individual participant data: rationale, conduct, and reporting BMJ 2010 340 c221 10.1136/bmj.c221 20139215

[B10] Stewart LA Clarke MJ Practical methodology of meta-analyses (overviews) using updated individual patient data. Cochrane Working Group Stat Med 1995 14 19 2057 2079 10.1002/sim.4780141902 8552887

[B11] Clark MJ Stewart LA Egger M, Davey-Smith GD, Altman DG Obtaining individual patient data from randomised controlled trials Systematic Reviews In Health Care Meta analysis in context 2001 London: BMJ Books

[B12] Abo-Zaid G Sauerbrei W Riley RD Individual participant data meta-analysis of prognostic factor studies: state of the art? BMC Med Res Methodol 2012 12 1 56 10.1186/1471-2288-12-56 22530717 PMC3413577

[B13] Lefebvre C Manheimer E Glanville J Higgins JPT, Green S Chapter 6 Searching for studieshttp://www.crd.york.ac.uk/prospero/ accessed 22/01/13

[B14] Hayden JA Pierre Cote DC Bombardier C Evaluation of the quality of prognostic studies in systematic reviews Ann Intern Med 2006 144 427 437 16549855 10.7326/0003-4819-144-6-200603210-00010

[B15] Crombie IK The Pocket Guide to Critical Appraisal 1996 London: BMJ publishing group

[B16] McShane LM Altman DG Sauerbrei W Taube SE Gion M Clark GM for the statistics subcommittee of the NCI-EORTC working group on cancer diagnostics J Natl Cancer Inst 2005 97 16 1180 1184 10.1093/jnci/dji237 16106022

[B17] Altman DG Lyman GH Methodological challenges in the evaluation of prognostic factors in breast cancer Breast Cancer Res Tr 1998 52 289 303 10.1023/A:1006193704132 10066088

[B18] Rector T Taylor BC Wilt TJ Chapter 12: systematic review of prognostic tests J Gen Int Med 2012 27 suppl S94 S101 10.1007/s11606-011-1899-y PMC3364355 22648680

[B19] Altman DG Systematic reviews of evaluations of prognostic variables BMJ 2001 323 7306 224 228 10.1136/bmj.323.7306.224 11473921 PMC1120839

[B20] Laupacis A Sekar N Stiell IG Clinical prediction rules. A review and suggested modifications of methodological standards J Am Med Assoc 1997 277 6 488 494 10.1001/jama.1997.03540300056034 9020274

[B21] Wasson JH Sox HC Neff RK Goldman L Clinical prediction rules. Applications and methodological standards New Engl J Med 1985 313 13 793 799 10.1056/NEJM198509263131306 3897864

[B22] Fowkes FRG Fulton PM Critical Appraisal of published research, introductory guidelines BMJ 1991 302 1136 1140 10.1136/bmj.302.6785.1136 2043787 PMC1669795

[B23] Vandenbroucke JP Von Elm E Altman DG Gotzsche PC Mulrow CD Pocock SJ Strengthening the Reporting of Observational Studies in Epidemiology (STROBE): explanation and elaboration PLoS Med 2007 4 10 e297 10.1371/journal.pmed.0040297 17941715 PMC2020496

[B24] Von Elm E Altman DG Egger M Pocock SJ Gotzsche PC Vandenbroucke JP The Strengthening the Reporting of Observational Studies in Epidemiology (STROBE) statement: guidelines for reporting observational studies J Clin Epidemiol 2008 61 4 344 349 10.1016/j.jclinepi.2007.11.008 18313558

[B25] Whiting PF Rutjes AWS Westwood ME Mallett S Deeks JJ Reitsma JB Leeflang MMG Sterne AV Bossuyt PMM QUADAS-2: A revised tool for the quality assessment of diagnostic accuracy studies Ann Intern Med 2011 155-529-536 10.7326/0003-4819-155-8-201110180-00009 22007046

[B26] Stroup DF Berlin JA Morton SC Ingram O Williamson CD Rennie D Moher D Becker BJ Sipe TA Thaker SB For the meta analysis of observaltional studies in epidemiology (MOOSE) group. Meta analysis of observational studies in epidemiology JAMA 2000 283 15 2008 12 10.1001/jama.283.15.2008 10789670

[B27] Systematic reviews CRDs Guidance for undertaking reviews in health care 2008 The University of York http://www.york.ac.uk/inst/crd/index_guidance.htm Accessed

[B28] Higgins JP Thompson SG Quantifying heterogeneity in a meta-analysis Stat Med 2002 21 11 1539 155827 10.1002/sim.1186 12111919

[B29] Royston P Multiple imputation of missing values: update Stata J 2005 5 188 201

[B30] Sterne JA White IR Carlin JB Spratt M Royston P Kenward MG Multiple imputation for missing data in epidemiological and clinical research: potential and pitfalls BMJ 2009 338 b2393 10.1136/bmj.b2393 19564179 PMC2714692

[B31] Steyerberg EW *Validation of prediction models* Clinical Prediction Models. A practical approach to development, validation and updating 2009 US: Springer

[B32] Medical Research Council (MRC) Ethics Series Personal Information in Medical Researchhttp://www.mrc.ac.uk (accessed 14th May 2012)

[B33] Moher D Liberati A Tetzlaff J Altman DG Preferred reporting items for systematic reviews and meta-analyses: the PRISMA statement Ann Int Med 2009 151 4 264 9 W64 19622511 10.7326/0003-4819-151-4-200908180-00135

[B34] PROSPERO International Prospective Register of Systematic Reviews 2012 NHS National Institute of Health

