# Supplementary material for: Protocol for a systematic review and individual patient data meta-analysis of prognostic factors of foot ulceration in people with diabetes: the international research collaboration for the prediction of diabetic foot ulcerations (PODUS)
Source: BMC Med Res Methodol. 2013 Feb 15;13:22. doi: 10.1186/1471-2288-13-22 (PMC3599337; doi:10.1186/1471-2288-13-22)
Supplement: Additional file 1: Appendix 1 — Flow diagram of the stages in an IPD review adapted from Stewart and Clark 199510. [file 1471-2288-13-22-S1.doc]

**APPENDIX 1. Flow Diagram - Stages of an Individual Patient-based Meta-analysis**[**^10^**](#_ENREF_7)

Identify need for IPD meta analysis

Identify need for IPD meta-analysis

Devise/refine questions

Development

Background Research

Identify studies

Meta-analysis of published data

Approx 3-6 months

Write protocol

Contact authors

Data Collection and Checking

Assess feasibility

Approx 12 months

Prepare data

Analysis of individual studies

Set up database

Check data

Finalise database

Analysis and dissemination of results

Analyse Data

Approx 3-6 months

Present Results

Draft Manuscript

**Make recommendations for research and clinical practice**
