# Supplementary material for: Protocol for a systematic review and individual patient data meta-analysis of prognostic factors of foot ulceration in people with diabetes: the international research collaboration for the prediction of diabetic foot ulcerations (PODUS)
Source: BMC Med Res Methodol. 2013 Feb 15;13:22. doi: 10.1186/1471-2288-13-22 (PMC3599337; doi:10.1186/1471-2288-13-22)
Supplement: Additional file 2: Appendix 2 — Embase and MEDLINE searches. [file 1471-2288-13-22-S2.doc]

**APPENDIX 2. Embase and MEDLINE searches**

**Embase Search**

1. Diabetic Foot/

2. (diabet$ and (foot or feet or toe$)).ti,ab.

3. 1 or 2

4. (foot or feet or toe$).ti,ab.

5. experimental diabetes mellitus/

6. insulin dependent diabetes mellitus/

7. Non Insulin Dependent Diabetes Mellitus/

8. diabetic neuropathy/ or neuropathy/

9. Diabetic Angiopathy/

10. or/5-9

11. Foot Ulcer/

12. exp vascular disease/

13. (foot ulcer$ or isch#em$ or vascular dis$).ti,ab.

14. amput$.ti,ab.

15. or/11-14

16. (vibration or touch or skin temperature$ or tuning fork$).ti,ab.

17. (monofilament$ or biothesiometer$ or ankle brachial ind$ or ultraso$).ti,ab.

18. skin temperature/

19. echography/ or doppler echography/

20. (screen$ or predict$ or sensitiv$ or specif$ or risk$ or assess$).ti,ab.

21. or/16-20

22. (3 or (4 and 10 and 15)) and 21

23. limit 22 to embase

**MEDLINE Search:**

1. diabet$.ti,ab.

2. (foot or feet or toe$).ti,ab.

3. Diabetes Mellitus, Experimental/

4. Diabetes Mellitus, Type I/

5. Diabetes Mellitus, Type II/

6. Diabetic Angiopathies/

7. Diabetic Foot/

8. Foot Ulcer/

9. Diabetic Neuropath$.mp. [mp=title, abstract, original title, name of substance word, subject heading word, protocol supplementary concept, rare disease supplementary concept, unique identifier]

10. Vascular Diseases/

11. Peripheral Vascular Diseases/

12. ISCHEMIA/

13. (foot ulcer$ or isch#em$ or vascular dis$).ti,ab.

14. amput$.ti,ab.

15. (vibration or touch or skin temperature$ or tuning fork$).ti,ab.

16. (monofilament$ or biothesiometer$ or ankle brachial ind$ or ultraso$).ti,ab.

17. Skin Temperature/

18. ULTRASONOGRAPHY/

19. Ultrasonography, Doppler/

20. or/10-14

21. or/15-19

22. 1 and 2

23. (or/3-6) or 9

24. 8 and (22 or 23)

25. 1 and 2 and 21

26. 7 and 21

27. 2 and 21 and 23

28. 21 and 24

29. or/25-28

30. (screen$ or predict$ or sensitiv$ or specific$ or risk factor$ or assess$).ti,ab.

31. (or/15-19) or 30

32. 1 and 2 and 30

33. 7 and 31

34. 2 and 23 and 31

35. 24 and 31

36. or/32-35
