# Supplementary material for: Protocol for a systematic review and individual patient data meta-analysis of prognostic factors of foot ulceration in people with diabetes: the international research collaboration for the prediction of diabetic foot ulcerations (PODUS)
Source: BMC Med Res Methodol. 2013 Feb 15;13:22. doi: 10.1186/1471-2288-13-22 (PMC3599337; doi:10.1186/1471-2288-13-22)
Supplement: Additional file 3: Appendix 3 — Questionnaire to determine the methodological standards adopted in cohort studies evaluating the prognostic factors for foot ulceration in diabetes. [file 1471-2288-13-22-S3.doc]

**APPENDIX 3. Data Extraction and Quality Assessment Checklist.**

**DATA EXTRACTION (Study Characteristics)**

| **Questions** | | **Details** |
| --- | --- | --- |
| Was the purpose of the study to derive or validate a model of prognostic factors for foot ulceration? | | Derive Yes/No  Validate Yes/No |
| **Methods** | | |
| Setting/context | Describe the setting (primary care, hospital, GP practice). |  |
|  | Who took the measurements? (Podiatrist, GP, nurse etc). |  |
|  | Geographical location |  |
|  | Year study was carried out |  |
|  | Document dates during which study was conducted for periods of;  Recruitment  Examination  Measurement  Follow-up. |  |
| Participants | Describe the eligibility criteria |  |

**APPENDIX 3. Data Extraction and Quality Assessment Checklist, (cont).**

**QUALITY ASSESSMENT (Risk of Bias)**

| Selection of patients | Was the selection of patients conducted in such a way to avoid bias? | Yes: a consecutive or random sample of patient with diabetes were recruited.  No: a consecutive or random sample of patient with diabetes were not recruited.  Unclear: no information about the manner in which patients were recruited is given. |
| --- | --- | --- |
| Timing of follow-up | Was the timing of follow-up long enough for an ulcer to develop? | Yes: The follow-up was conducted at least one month after the baseline tests were completed.  No: The follow-up was conducted within one month after the baseline tests were completed.  Unclear: The timing of the follow-up is not known |
| Replicating the tests | Is there sufficient explanation of the conduct of the tests to permit their replication? | Yes: The conduct of each test can be replicated from the explanation.  No: It is not possible to replicate the conduct of each test from the explanation.  Unclear: no information about the test conduct exists. |
| Blinding | Were the investigators who collected the follow-up data blind to the results of the index test? | Yes: The follow-up was conducted by investigators who were unaware of  the results of the index test.  No: The follow-up was conducted by investigators who knew the results of the index test.  Unclear: no information about the follow-up exists. |
| Study size | Has the study size been explained in detail? | Yes: A sample size calculation to justify the study size is available  No: A sample size calculation to justify the study size is unavailable  Unclear: no information about the follow-up exists. |
| **Results** | | |
| Participants | Is a flow diagram available showing the numbers of individuals at all stages of the study, numbers potentially eligible, who were examined for eligibility, were included in the study, and completed follow-up and outcomes). | Yes: a flow diagram showing the numbers of individuals at all stages of the study, the numbers potentially eligible, who were examined for eligibility, were included in the study, and completed follow-up and outcomes exists.  No: a flow diagram showing the numbers of individuals at all stages of the study, the numbers potentially eligible, who were examined for eligibility, were included in the study, and completed follow-up and outcomes does not exist. |
