# Supplementary material for: Protocol for a systematic review and individual patient data meta-analysis of prognostic factors of foot ulceration in people with diabetes: the international research collaboration for the prediction of diabetic foot ulcerations (PODUS)
Source: BMC Med Res Methodol. 2013 Feb 15;13:22. doi: 10.1186/1471-2288-13-22 (PMC3599337; doi:10.1186/1471-2288-13-22)
Supplement: Additional file 4: Appendix 4 — List of the most common variables reported in cohort studies. [file 1471-2288-13-22-S4.doc]

**APPENDIX 4. List of the most common variables reported in cohort studies**

| - Age - Sex - BMI - Socio-economic status - Educational level - Alcohol consumption - Smoking habits - Diabetes type - Diabetes duration - Diabetes therapy (eg Diet alone, insulin dependent, oral and hypoglycaemic drugs) - Previous ulcer - Previous lower limb amputation - Physical impairments - Peripheral Arterial Disease - Intermittent Claudication - History of Myocardial infarction - History of stroke - History of kidney disease - Malignancy | **Blood tests (and date recorded):**   - Plasma glucose (HbA1c) - Serum glycosylated haemoglobin - Serum creatinine - Erythrocyte sedimentation rate - Serum A1c - Serum cholesterol/total - HDL cholesterol - Total cholesterol - Uric acid - Any other blood tests   **Medication**   - Prescribe medication   **Outcomes**   - Death - Cause of death - Date of death - Time to death (days) - Incident ulcer/ulcer development - Date of ulcer onset - Time to ulcer/follow-up - End of follow-up |
| --- | --- |
| **Information on feet such as:**   - Hammer/claw toe - Charcot (deformity) - Prominent metatarsic heads - Abnormal foot shape - Callus - Body prominences - Hallux limitus - Hallux valgus - Ankle mobility - Limited Joint motion - Limited ROM subtalar joint - Hallux mobility (Hallux rigitus) - Tinea pedis - Pedal edema - Onchomyosis - Footwear :Type of footwear and type of socks - Symptoms:   - Foot numbness   - Foot pain   - Any other information - Signs (foot care habits):   - Good nail care   - Moisturised skin   - Any other information - Oedema   **Sight:**   - Visual impairment/ Eye problems due to diabetes - Has the patient had laser photocoagulation? | **Tests carried out:**   - Semmes Weinstein Monofilament (SWF) - Vibration Perception Threshold (VPT) - Neuro/biothesiometer   - Tuning fork - Neurological tests:   - Achilles tendon reflex   - TcP02 dorsal foot (mmHg)   - Doppler test   - Any other tests   - Information collected on neuropathy/ neuropathy symptoms   - Tendon reflex: - Blood pressure:   - Bp   - Hallux Bp   - Ankle Arm Index (AAI or Ankle Brachial Index ABI)   - Incompressible ankle pressure - Pulses:   - Peripheral pulses   - Any missing pulse - Information collected on peak plantar foot pressure |
