# Supplementary material for: Protocol for a systematic review and individual patient data meta-analysis of prognostic factors of foot ulceration in people with diabetes: the international research collaboration for the prediction of diabetic foot ulcerations (PODUS)
Source: BMC Med Res Methodol. 2013 Feb 15;13:22. doi: 10.1186/1471-2288-13-22 (PMC3599337; doi:10.1186/1471-2288-13-22)
Supplement: Additional file 5: Appendix 5 — Committees and members. [file 1471-2288-13-22-S5.doc]

Appendix 5

Project Committee Structure

Individual Patient Data meta-analyses are undertaken by collaborative groups. This systematic review has three distinct committees to ensure the research objectives are delivered as efficiently as possible. They each have different roles and responsibilities:

The Research Management Committee

will over-see the methodological development and the attainment of project milestones. They also have responsibility for reporting the progress to the NHIR HTA within the standard reporting mechanisms required by the HTA Clinical Evaluation and Trials Board.

The Data Management Committee

is a small group of epidemiologists, health services researchers and statisticians who are directly responsible for the data collection, assessment, cleaning and preparation for the meta analysis. Only these individuals will have access to the data from individual studies during the research. They will work closely with individual members of the International Steering Committee while preparing data from individual studies.

The International Steering Committee

The principal investigators of previously published cohort studies have agreed to take part and contribute the data from their study. Together they possess all data in existence.

We have strengthened the clinical and methodological input from five additional members who possess clinical and methodological expertise in diabetic medicine, foot care in primary and community settings, clinical prediction rules and IPD meta-analyses.

The collaboration of these clinical and methodological experts will help ensure the analysis is relevant to routine clinical practice in a variety of health care settings. All collaborators will contribute to and participate in the interpretation of the results during a face to face meeting in Edinburgh. Importantly they will help the dissemination of the research findings into routine NHS clinical practice.

All members from these committees are collectively known as “The Collaborators” who will be entitled to be named authors on journal outputs from the research. It is anticipated that there will be a group name used for publication e.g. International collaboration for clinical prediction rules used in the risk assessment of diabetic foot ulceration.

Members of the international collaboration for research into the prediction of foot ulcerations in diabetes:

Fay Crawford, Chantelle Anandan, Francesca M Chappell, Gordon D Murray, Jaqueline F Price, Aziz Sheikh, Colin R Simpson, Gerard P Stansby, Matthew J Young, Caroline A Abbott, Andrew JM Boulton, Edward J Boyko, Thomas Kastenbauer, Graham P Leese, Martin Maxwell (public partner) Matteo Monami, Matilde Monteiro-Soares, Stephen J Rith-Najarian, Aristidis Veves, Nikki Coates, William J Jeffcoate, Nicola Leech, Tom Fahey, Jayne Tierney.
