# Supplementary material for: Protocol for a systematic review and individual patient data meta-analysis of prognostic factors of foot ulceration in people with diabetes: the international research collaboration for the prediction of diabetic foot ulcerations (PODUS)
Source: BMC Med Res Methodol. 2013 Feb 15;13:22. doi: 10.1186/1471-2288-13-22 (PMC3599337; doi:10.1186/1471-2288-13-22)
Supplement: Additional file 6: Appendix 6 — Data agreement for the collaborators. [file 1471-2288-13-22-S6.doc]

**APPENDIX 6. Data confidentiality agreement**

**Individual patient data for cohort studies**

Cohort study datasets (individual patient data) will be supplied by the collaborators directly to members of the Data Management Committee. All cohort data will be anonymised by the collaborators before it is dispatched.

**Ethics and governance**

This study does not require ethical committee approval for the following reasons:

1. Investigators of the original studies obtained local ethical committee approval and written, informed patient consent;
2. The project seeks anonymised data from which the individuals recruited to the original study cannot be identified

**Confidentiality, data storage, access and archiving**

Anonymised datasets from each of the collaborators of the primary cohort studies will be provided in a manner deemed most convenient to them (for example on encrypted USB sticks).  Data will be stored in password protected files on a secure University of Edinburgh computer [University of Edinburgh Data protection registration number: Z6426984] and will only be accessible by members of the Data Management Committee. The anonymised datasets and final Individual Patient Dataset will be deposited in a data archive in accordance with NHS procedures for data archiving.

**Use of the data**

Data will be used only in the agreed manner detailed in this protocol. Any additional analyses will require the approval of the international collaborators.

**Research Governance Framework**

Any research connected with this project will be in accordance with the Department of Health Guidance “Research Governance Framework for Health and Social Care”.

| I agree to supply those data listed in Appendix 4 of this protocol that are in my possession in an anonymised format ensuring no patient identity is revealed and confirm that local ethics approval was obtained prior to patient recruitment of the original study:  Collaborator name __________________________________ Date___________ |
| --- |

I confirm that all data will be stored in secure password-protected files only accessible to members of the data management committee and these will ultimately be archived in accordance with the patient data archiving procedures required by the National Health Service (NHS). All data will be used for analysis according to the plan outlined in this protocol.

Professor Gordon Murray
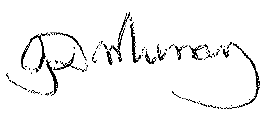
Murray Date

Dr Fay Crawford
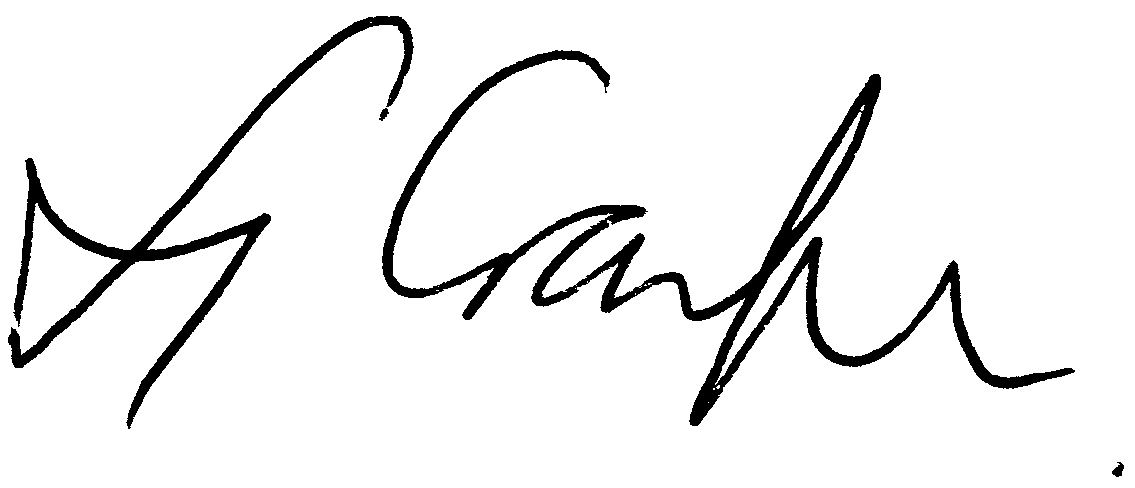
Date
